# Diabetes mellitus among patients attending TB clinics in Dar es Salaam: a descriptive cross-sectional study

**DOI:** 10.1186/s12879-019-4539-5

**Published:** 2019-10-29

**Authors:** Patricia J. Munseri, Henrika Kimambo, Kisali Pallangyo

**Affiliations:** Department of Internal Medicine, Muhimbili University of Health and Allied Sciences Dar es Salaam, Box 65315, Dar es Salaam, Tanzania

**Keywords:** Diabetes mellitus, Tuberculosis, Screening, Routine care, Tanzania

## Abstract

**Background:**

A bi-directional interaction between diabetes mellitus and tuberculosis is well established and has been likened to that between HIV and TB. Whereas HIV screening is standard of care test in sub Saharan Africa TB programs, the same is not true for diabetes mellitus (DM). Sub Saharan Africa, a region with high TB infection rates, is going through an epidemiological transition with rapidly rising prevalence of diabetes.

We aimed at characterizing TB patients with DM in order to identify factors associated with TB-DM dual disease among patients attending TB clinics in Dar es Salaam.

**Methods:**

A cross-sectional study was conducted between September 2016 and January 2017 among patients attending TB clinics in Dar es Salaam. We collected socio-demographic characteristics, anthropometric measurements and screened for diabetes by measuring fasting blood glucose that was followed by a 2 h postprandial glucose for participants with impaired fasting blood glucose. We examined for socio-demographic and clinical factors associated with diabetes using logistic regression analysis.

**Results:**

Of the 660 enrolled participants with TB, 25 (3.8%) were on treatment for diabetes while 39 (6.1%) and 147 (23%) of the remaining 635 participants were ultimately diagnosed with DM and impaired fasting blood glucose respectively. The overall prevalence of DM was 9.7% (64/660). Independent risk factors for diabetes included: age > 44 years {OR 4.52, 95% CI: [1.28–15.89]}; family history of diabetes {OR 3.42, 95% [CI 1.88–6.21]}.

HIV sero-positive TB patients were less likely to have DM compared to those who were HIV sero-negative {OR 0.35, 95% CI [0.17–0.73]}.

**Conclusions:**

Screening for diabetes should be advocated for TB patients aged above 44 years and/or with a family history of diabetes. HIV sero-negative TB patients were more likely to have DM compared to those who were HIV sero-positive. Further studies are needed to confirm this observation and the underlying factors.

## Introduction

The prevalence of diabetes worldwide has increased by about 20% in the past three decades with particularly large rates in low and middle income countries [1, 2]. The International Diabetes Federation (IDF) predicts further increase in prevalence of DM especially in developing countries in the foreseeable future due to changes in life style, eating habits and other risk factors [3]. Indeed countries in sub Saharan Africa (SSA), are going through an epidemiological transition with rising prevalence of non-communicable diseases including type two DM (T2DM) [4]. For example between the 1980s and 2010 the prevalence of DM in rural Tanzania increased from 0.9 to 5% [5, 6]. Furthermore data from the city of Dar- es -salaam indicates that the prevalence of DM among the general population increased from < 2 to 9.1% during the period of 1980–2012 [5, 6]. A bi-directional interaction between DM and Tuberculosis is well established [7]. People with DM are three times at risk of developing TB disease compared to non-diabetics [8]. Glucose intolerance or transient hyperglycemia has been observed in up to 49% of patients with active TB [9]. Patients with DM have impaired cellular immunity and ciliary function that predisposes them to TB while stress response to TB may result to insulin resistance. TB disease of the pancreas can lead to endocrine hypo-function with consequent DM [7]. In addition DM is an independent risk factor for poor TB treatment response as well as death [10, 11]. In 2015 the IDF reported that 66.7% of people with diabetes in SSA are unaware that they have the disease [3].

Tanzania is a high TB endemic country with reported prevalence rates of 293 cases per 100,000 for individuals aged 15–64 years and 709 cases per 100,000 for individuals above 65 years of age [12]. Hence the rising prevalence of DM is likely to have significant negative impact on TB control and has been likened to the impact of HIV on TB control [13].

Consequently, screening TB patients for DM and vice versa should result in better control of both diseases, as this would aid in early detection and treatment that would result in better outcomes. Indeed, routine screening of TB patients for DM using HbA1c in India by Balachrishnan et al. found that over 40% had diabetes [14]. However, Balachrisnan and colleagues did not advocate routine DM screening using HbA1c but recommended further operational research be performed to determine the most cost- effective ways of diabetes screening.

We aimed at determining the prevalence of DM among TB patients and characterizing TB patients with DM in order to identify factors associated with TB-DM dual disease among patients attending TB treatment clinics in Dar es Salaam, Tanzania.

## Methods

### Ethics statement

Ethical approval was obtained from the Muhimbili University of Health and Allied Science institutional review board approval number MU/PGS/SAEC/Vol.XVI/. All recruited participants were informed about the study details prior to enrollment and verbal consent was obtained from each study participant prior to enrollment in the study. Patients who were newly diagnosed with diabetes during this study were referred to a diabetic clinic to receive standard of care treatment for diabetes.

### Study design and population

This descriptive cross-sectional study was conducted in four TB clinics in Dar es Salaam, Tanzania from September 2016 to January 2017. Each clinic represents one of the three districts in Dar es Salaam and receives about 30 TB patients per day. Consecutive patients on treatment for tuberculosis aged 18 years or above attending the four clinics at the time of the study were eligible and consenting patients were enrolled into the study. Recruited participants were either newly diagnosed TB patients or patients who were continuing with TB treatment.

### Data collection

Data was collected using an interviewer based structured questionnaire. A research assistant conducted face to face interviews and physical examination. Information collected included socio-demographic characteristics, symptom screening for diabetes, factors associated with type 2 diabetes including alcohol consumption, cigarette smoking and a past medical history related to diabetes. Smoking was categorized as ever smoked in a lifetime or never smoked, Alcohol intake was categorized as ever taken alcohol or never taken alcohol in their lifetime.

We also collected information on sputum smear status, TB anatomical site, TB treatment category, and HIV status from the participants TB treatment cards.

We measured the participant’s weight and height using standard equipment based at the TB clinics thereafter computed body mass index (BMI). BMI was computed as the participant’s weight in kilograms divided by the participant’s height in meters squared. BMI was grouped into four categories as follows; BMI of < 18.5 was regarded as underweight, 18.5–24.9 normal weight, 25.0–29.9 overweight and a BMI > 20.9 was regarded as obese [15, 16].

A capillary fingertip blood sample was collected from each patient for fasting blood glucose. All participants with impaired fasting blood glucose were given 75 mg of oral glucose that was followed by a two-hour post-prandial blood glucose measurement using standard ACCU-CHECK blood glucose meter. The American Diabetic Association (ADA) diagnostic criteria were used for diagnosis of diabetes [17]. Diabetes was defined as either a fasting blood glucose level of > 6.9 mmol/L or a 2-h postprandial glucose level of ≥11.1 mmol/L while impaired fasting glucose was defined as glucose levels of 5.6 mmol/L- 6.9 mmol/L [18].

### Statistical methods

The total number of participants recruited to estimate the prevalence of diabetes was based on the sample size estimation formula with an estimated prevalence of diabetes among TB patients to be 16.7% [19], type I error at 5 and 3% precision that amounted to 594 participants.

Data was transferred from the questionnaires and entered into SPPS version 23.0 database for cleaning and analysis. Continuous variables were summarized and presented as mean and standard deviation while categorical variables were summarized as proportions. Univariate and multivariate logistic regression analysis was performed to examine for factors that were associated with the outcome (diabetes mellitus) using Pearson’s chi square test. All covariates with a *p* value of < 0.2 in univariate analysis, and confounders such as age, were entered in the multivariate analysis model based on literature [20]. As the symptoms for diabetes such as polyuria, nocturia, polyphagia and polydipsia were correlated we summarized these factors into one composite variable that was labeled any symptom for diabetes. Statistical significance was set at a *p* value < 0.05. For the multivariate and univariate model, risks were calculated and summarized as odds ratios and 95% confidence intervals significance level was set as a *p* value of < 0.05 in the multivariate analysis model.

## Results

### Socio-demographic and clinical characteristics

During the study period we recruited 660 participants out of 678 who were on TB treatment, 18 (2.6%) of the participants withheld their consent, 416 (63%) were males. The mean age and standard deviation of the study population was 39.2 ± 14.3 years and the mean body mass index was 21.0 kg/m^2^. One hundred and sixteen (17.6%) participants had a positive family history of DM and 25 (3.8%) participants were on treatment for diabetes mellitus. Other socio-demographic characteristics are summarized in Table 1.

**Table 1:** Socio-demographic characteristics of study participants (*N* = 660)

Characteristic	n	%
Sex		
Male	416	63
Female	244	37
Age groups (years)		
≤ 24	87	13.2
25–34	181	27.4
35–44	172	26.1
≥ 45	220	33.3
Highest education level		
No formal education	65	9.8
Primary	430	65.2
Secondary	121	18.3
College	44	6.7
Marital Status		
Single	188	28.5
Married	355	53.8
Divorced	117	17.7
Occupation		
Unemployed	145	22
Employed	484	73.3
Retired	31	4.7
Family history of diabetes		
Yes	116	17.6
Cigarette smoking		
Ever smoked	180	27.3
Alcohol use		
Ever used	267	40.5

A total of 212 (32%) participants had TB/HIV co-infection and 191 (90%) of them were on HIV treatment. Of the enrolled participants 577 (87.4%) had pulmonary tuberculosis and 389 (60.3%) were smear positive for acid-fast bacilli as shown in Table 2.

**Table 2:** Clinical characteristics of study participants (*N* = 660)

Characteristic	n	%
Body mass Index Kg/M^2^		
Underweight	173	26.3
Normal	398	60.4
Missing (*n* = 1)^a^		
Overweight	66	10.0
Obesity	22	3.3
Polyuria	110	16.7
Polyphagia	68	10.3
Polydipsia	82	12.4
Nocturia	151	22.9
Loss of weight	479	72.6
Fatigue	375	56.8
Blurred vision	100	15.2
Fasting blood glucose levels in mmol/L (n-635)		
Normal	453	71.3
Impaired fasting glucose	147	23.1
Diabetes	35	5.5
Oral glucose tolerance test (*n* = 147)		
Normal	110	74.1
Impaired glucose tolerance	33	23.1
Diabetes	4	2.8
Sputum Acid fast bacilli status		
Positive	398	60.3
Tuberculosis anatomical site		
Pulmonary	577	87.4
TB treatment category		
First line	578	87.6
First line re-treatment	68	10.3
Second line	14	2.1
Tuberculosis treatment phase		
Intensive	269	40.8
Continuation	391	59.2
HIV sero-status		
Positive	212	32.1
On HIV treatment		
Yes	191	90.1

^a^Height could not be measured, as patient was unable to stand upright

### Prevalence of diabetes mellitus

**Fig. 1:**
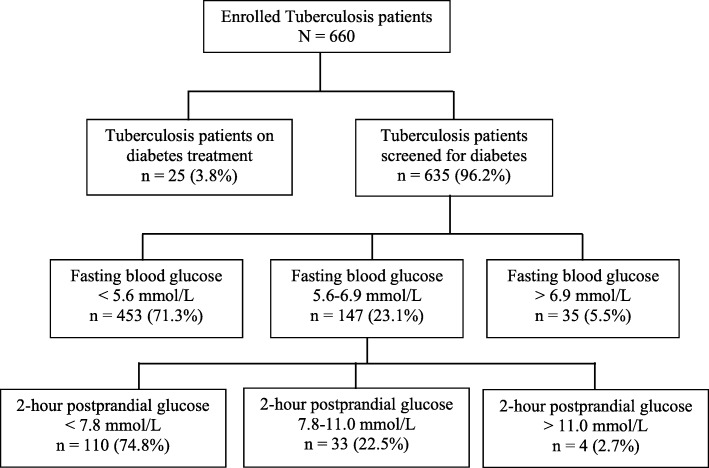
Consort diagram showing the flow of participants

A consort diagram Fig. 1, summarizes the flow of study participants. Of the 660 enrolled study participants with TB, 25 (3.8%) were on treatment for DM and therefore the remaining 635 (96.2%) participants were screened for DM. Out of the 635 participant who were screened 35 (5.5%) were diagnosed to have DM with a fasting blood glucose of > 6.9 mmol/L and 147 (23.1%) had an impaired fasting blood glucose (fasting blood glucose between 5.6–6.9 mmol/L). The 147 study participants with impaired fasting blood glucose underwent a two-hour post-prandial oral glucose tolerance test, 4 (2.7%) were diagnosed with diabetes and 33 (22.5%) had impaired oral glucose tolerance test. Therefore, the overall prevalence of DM among the study participants with TB was 9.7% (64/660), 95% CI (7.4–12.0%) while the prevalence of newly diagnosed DM among the study participants was 6.1%. Impaired fasting blood glucose was detected in 23.1% (147/635) 95% CI (19.6–26.6).

### Factors associated with DM in TB patients

Table 3 summarizes the factors associated with DM, age of > 44 years was associated with a higher prevalence (15.9%) of diabetes among TB patients (*p* < 0.001). and a family history of diabetes mellitus was significantly associated with a higher prevalence of DM (20.7% vs 7.4%) *p* < 0.001. DM was more prevalent in TB patients who were overweight and obese each accounting to 18% compared to patients who were underweight 7.5% or of normal weight 8.5% (*p* = 0.031). Among the 64 diabetic patients with TB, 55 (9.5% were categorized as new TB cases, 7 (10.8%) were relapse TB and 2 (14.3%) were drug resistant TB. There was no association between DM and clinical type of TB or results of sputum smear for acid-fast bacilli (data not shown).

**Table 3:** Factors associated with diabetes

Characteristic	Diabetes *n* = 64	Total	*P*-value
Sex			
Male	43 (10.3%)	416 (63.0%)	
Female	21 (8.6%)	244 (37%)	0.499
Age group in years			
≤ 24	3 (3.4%)	87 (13.2%)	
25–34	10 (5.5%)	181 (27.4%)	
35–44	16 (9.3%)	172 (26.1%)	0.001
> 44	35 (15.9%)	220 (33.3%)	
Marital status			
Never married	11 (5.9%)	188 (28.5%)	
Married	43 (12.1%)	355 (53.8%)	0.057
Divorced	10 (8.5%)	117 (17.7%)	
Education level			
No formal education	3 (4.6%)	65 (9.8%)	
Primary	47 (10.9%)	430 (65.2%)	0.32
Secondary	9 (7.4%)	121 (18.3%)	
College	5 (11.4%)	44 (6.7%)	
Occupation			
Unemployed	11 (7.6%)	145 (22.0%)	
Employed	49 (10.1%)	484 (73.3%)	0.708
Retired	4 (12.9.3%)	31 (4.7%)	
Family history of diabetes			
Yes	24 (20.7%)	116 (17.6%)	
No	40 (7.4%)	544 (82.4%)	< 0.001
Cigarette smoking			
Ever smoked	18 (10.0%)	180 (27.3%)	
Never smoked	46 (9.6%)	480 (72.7%)	0.69
Alcohol use			
Ever used	26 (9.7%)	267 (40.5%)	
Never used	38 (9.7%)	393 (59.5%)	0.791
Body mass Index Kg/M^2^			
Underweight	13 (7.5%)	173 (26.3%)	
Normal	34 (8.5%)	398 (60.4%)	0.031
Overweight	12 (18.2%)	66 (10.0%)	
Obesity	4 (18.2%)	22 (3.3%)	
HIV sero-status			
Negative	53 (11.8%)	448 (68%)	
Positive	11 (5.2%)	212 (32%)	0.007

### Predictors for DM in patients with TB

The predictors for diabetes in patients with TB were as summarized in Table 4. In univariate analysis factors that were associated with an increased risk for diabetes were: any symptom for diabetes (polyphagia, polydipsia, polyuria or nocturia) {OR 2.25 (95% CI: 1.33–3.18)}, blurring of vision {OR 2.45 (95% CI: 1.36–4.44)}, a family history of diabetes {OR 3.29 (95% CI: 1.89–5.71)} and overweight {OR 2.73 (95%CI: 1.18–6.33)}. HIV infection was associated with a decreased risk for diabetes in TB patients {OR 0.41 (CI: 0.21–0.79)}.

**Table 4:** Predicators for diabetes among TB patients

Predictor	Univariate Analysis Odds Ratio (95 CI)	*P* value	Multivariate Analysis Odds Ratio (95 CI)	*P* value
Sex				
Female	1		1	
Male	1.22 (0.71–2.12)	0.469	1.07 (0.58–1.97)	0.837
Age				
< 24	1		1	
25–34	0.19 (0.06–0.63)	0.007	1.59 (0.41–6.15)	0.500
35–44	0.31 (0.15–0.64)	0.002	3.22 (0.87–11.94)	0.080
> 44	0.54 (0.29–1.02)	0.056	4.52 (1.28–15.89)	0.018
Any DM symptom				
No	1		1	
Yes	2.25 (1.33–3.18)	0.003	1.89 (1.05–3.42)	0.034
Weight loss				
No	1			
Yes	1.05 (0.59–1.88)	0.871		
Fatigue				
No	1			
Yes	1.29 (0.76–2.21)	0.335		
Blurred Vision				
No	1		1	
Yes	2.45 (1.36–4.44)	0.003	1.60 (0.82–3.13)	0.170
Family history				
No	1		1	
Yes	3.29 (1.89–5.71)	< 0.001	3.42 (1.88–6.21)	< 0.001
Ever Smoked				
No	1			
Yes	1.05 (0.59–1.86)	0.872		
Alcohol consumption				
No	1			
Yes	1.01 (0.59–1.70)	0.977		
Body Mass Index				
Underweight	1		1	
Normal	1.15 (0.59–2.24)	0.681	0.87 (0.43–1.75)	0.689
Overweight	2.73 (1.18–6.33)	0.019	1.73 (0.69–4.33)	0.242
Obesity	2.73 (0.81–9.28)	0.107	2.15 (0.57–8.03)	0.256
HIV sero-status				
Negative	1		1	
Positive	0.41 (0.21–0.79)	0.009	0.35 (0.17–0.73)	0.005

After controlling for other factors, age > 44 years {OR 4.52 (95% CI: 1.28–15.89)}, any symptom for diabetes {OR 1.89 (95% CI: 1.05–3.42)} and a family history of diabetes {OR 3.42 (95%: 1.88–6.21)} were associated with an increased risk for DM. While HIV infection {OR 0.35 (95% CI 0.17–0.73)} seemed to decrease the risk of diabetes in TB patient.

## Discussion

This descriptive cross-sectional study was conducted among patients attending routine clinics for TB, in Dar es Salaam. We found that about 10% of patients receiving TB treatment had DM which is higher than the estimated prevalence of 3.5 and 4.3% among adults aged 20–79 years in the general population [3, 21]. Similar prevalence rates of DM among TB patients have been reported in recent studies conducted in sub Saharan Africa [22–27] and elsewhere. Indeed, a review of several studies and a meta-analysis study on screening for DM among TB patients concluded that DM was associated with increased risk of TB [8].

Noteworthy however, is the fact that two thirds (60%) of our study participants with DM did not know they had diabetes (diagnosis of DM was a result of this study). These findings are in keeping with reports from several other studies [4, 21, 27, 28] and lend support to the recommendation that TB patients be screened for diabetes as T2DM is often asymptomatic and frequently presents initially with complications. Indeed, reports from the IDF show that more than two thirds of adults with T2DM are not aware they have the disease [3]. In spite of these reports, DM screening is not standard of care among TB patients in most settings in sub Saharan Africa and elsewhere.

Findings from this study show that TB patients aged 44 years and above had four-fold increased risk of diabetes. Likewise, TB patients with family history of DM had a threefold increased risk of diabetes. Findings similar to these have been reported elsewhere [24]. Our study findings do not advocate DM screening for all TB patients but instead suggest that TB patients aged 44 years and above and those with positive family history, should be screened for diabetes mellitus.

Untreated DM is known to be a risk factor for poor TB outcomes that include treatment failure, relapse and death. In addition, published data indicate that diabetic patients are likely to remain sputum smear positive for AFB for 2 to 3 months following treatment for TB [29]. In another study 22% of the diabetic TB patients remained sputum culture positive after 6 months treatment course with TB medications [22].

Screening of TB patients for DM should therefore identify patients who need extra attention and care for better TB treatment outcomes and prevention of complications related to untreated DM.

Over 23% of TB patients in this study had impaired fasting glucose (IFG) for which we are not able with certainty to explain the cause. However, IFG may be indicative of stress- induced hyperglycemia that would disappear after TB treatment [30–32] but it may also be indicative of high risk to developing DM.

We did not follow up the study participants with impaired glucose tolerance to determine if the impaired glucose tolerance detected was TB induced hyperglycemia or was an indicator of later development of DM. Due to the study design we are not able to establish what started first if it was DM or TB. Nonetheless, results of this study indicate that about a quarter (23%) of TB patients had IFG that may have been stress-induced, by the TB disease or an indicator of developing DM in the future. Such patients would benefit from regular blood sugar monitoring to detect early onset DM as well as appropriate changes in their lifestyle to prevent/delay onset of DM.

We observed that HIV sero-negative TB patients were more likely to have DM compared to those who were HIV sero-positive. Similar findings have been reported in other studies in SSA [19, 26, 33]. However, published reports on association between HIV and diabetes have not been consistent. A nationwide population based cohort study in Denmark found that HIV infected people not on HAART were not at increased risk of developing DM [34]. On the other hand a study from the USA reported increased risk of DM among HIV infected persons on treatment [35]. A meta-analysis on the relationship between HIV and DM in SSA concluded that there was no association between the two conditions [36].

Further studies are needed to examine the effects of CD4 cell count level HIV antiretroviral regimens, and obesity among TB HIV co-infection and DM.

There was a trend that indicated that overweight and obese individuals had an increased risk for development of DM, however due to small numbers of participants with overweight and obesity statistical significance could not be achieved.

The results of this study indicate that screening for DM among patients attending public TB clinics in Dar es Salaam was possible and led to detection of patients with unknown DM and impaired glucose intolerance. Furthermore, DM screening should be targeted to TB patients aged 44 years or more and those with a family history of DM. Nonetheless, similar studies need to be done to confirm our findings before policy change can be considered.

## Conclusion

Diabetes mellitus is a common co-morbidity in TB patients. Integration of diabetes screening particularly in TB patients aged 44 years and above and/or in patients with a family history of DM at TB clinics offers an opportunity for screening of patients who may have not otherwise presented to medical care. HIV sero-negative TB patients were more likely to have DM compared to those who were HIV sero-positive. Further studies are needed confirm this observation and the underlying reasons.

## Data Availability

Dataset used for analysis in this study are not publicly available, data are however available from the corresponding author on reliable request.

## Abbreviations

ADA: American diabetic association
AFB: Acid fast Bacilli
BMI: Body mass index
CD4: Cluster for differentiation
CI: Confidence intervals
DM: Diabetes mellitus
HAART: Highly active antiretroviral therapy
HIV: Human immunodeficiency virus
IDF: International Diabetes Federation
IFG: Impaired fasting glucose
SSA: Sub-Saharan Africa
T2DM: Type 2 diabetes mellitus
TB: Tuberculosis
